# Supplementary material for: The Efficacy of Polydioxanone Sutures in Treating Mild-to-Moderate Knee Osteoarthritis: A Systematic Review and Meta-Analysis
Source: Medicina (Kaunas). 2025 Feb 24;61(3):388. doi: 10.3390/medicina61030388 (PMC11944242; doi:10.3390/medicina61030388)
Supplement: Supplementary file 1 [file medicina-61-00388-s001.zip › Supplementary Figure S1. Risk of bias summary_24Feb2025.pdf]

Supplementary Figure S1. Risk of bias summary

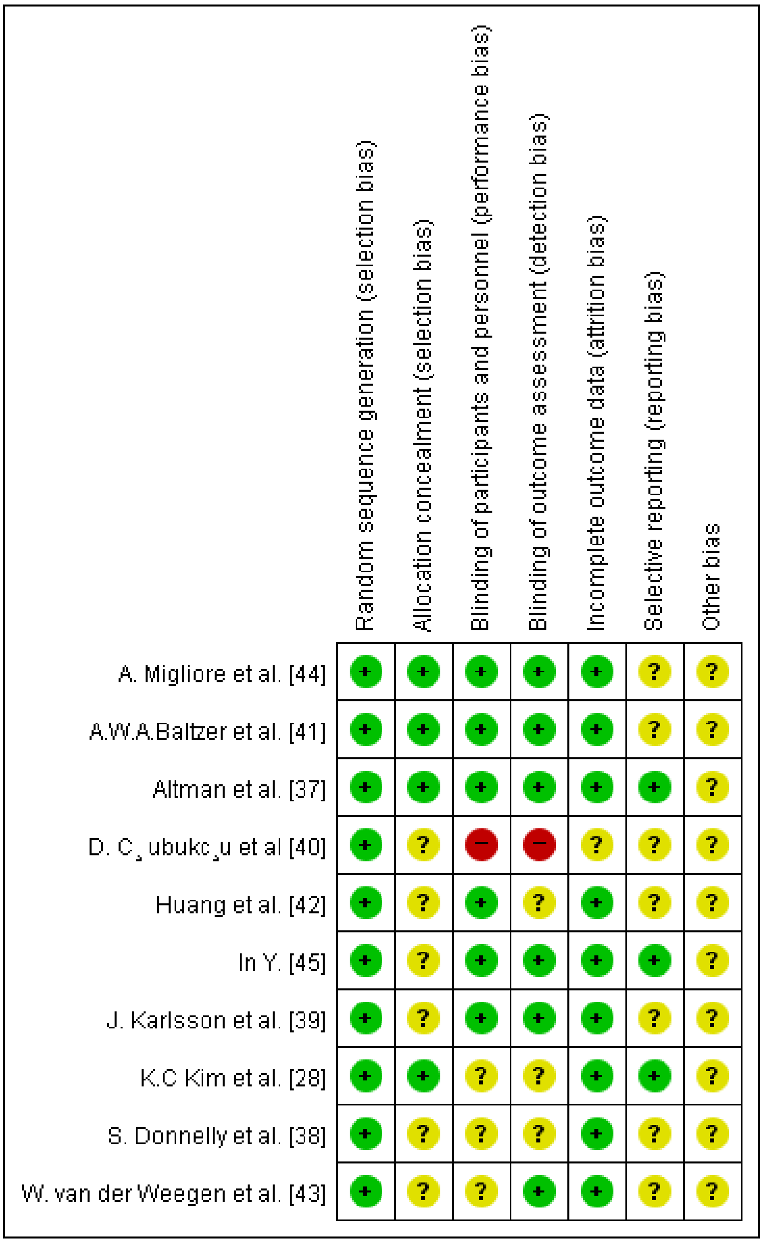

Review author's judgements for each risk of bias item for each included study. (+): Low risk of bias, (-): High risk of bias, (?): Unclear risk of bias [39]
