# Supplementary material for: The Efficacy of Polydioxanone Sutures in Treating Mild-to-Moderate Knee Osteoarthritis: A Systematic Review and Meta-Analysis
Source: Medicina (Kaunas). 2025 Feb 24;61(3):388. doi: 10.3390/medicina61030388 (PMC11944242; doi:10.3390/medicina61030388)
Supplement: Supplementary file 1 [file medicina-61-00388-s001.zip › Supplementary Table S2. Search strategies.pdf]

Supplementary Table S2. Search strategies

Database	Search terms	
MEDLINE	(("sodium hyaluronate"[Title/Abstract] OR "hyaluronate acid"[Title/Abstract] OR "Hyaluronate"[Title/Abstract] OR "hyaluronan"[Title/Abstract] OR "Viscosupplementation"[Title/Abstract] OR ("polynucleotide"[Title/Abstract] AND "pn"[Title/Abstract]) OR "Polydioxanone"[Title/Abstract]) AND (("Osteoarthritis"[Title/Abstract] OR "Osteoarthrosis"[Title/Abstract] OR "arthritis"[Title/Abstract] OR "arthrosis"[Title/Abstract] OR "Gonarthritis"[Title/Abstract] OR ((("knee"[MeSH Terms] OR "knee"[All Fields] OR "knee joint"[MeSH Terms] OR ("knee"[All Fields] AND "joint"[All Fields]) OR "knee joint"[All Fields]) AND "near"[All Fields]) AND "arthralgia"[Title/Abstract])) AND ("visual analogue scale score"[Title/Abstract] OR "vas"[Title/Abstract] OR "weight bearing pain"[Title/Abstract]) AND ("sham"[Title/Abstract] OR "placebo"[Title/Abstract] OR "saline"[Title/Abstract]))) AND ((ffrft[Filter]) AND (medline[Filter]) AND (clinicaltrial[Filter] OR meta-analysis[Filter] OR randomizedcontrolledtrial[Filter] OR systematicreview[Filter]) AND (humans[Filter]) AND (1000/1/1:2024/4/30[pdat]) AND (english[Filter] OR korean[Filter]) AND (alladult[Filter]))	
EMBASE	#11	#9 AND ('arthralgia'/dm OR 'knee osteoarthritis'/dm OR 'knee pain'/dm OR 'osteoarthritis'/dm OR 'pain'/dm) AND [adult]/lim AND 'article'/sd NOT [30-04-2024]/sd
	#10	#9 AND ('arthralgia'/dm OR 'knee osteoarthritis'/dm OR 'knee pain'/dm OR 'osteoarthritis'/dm OR 'pain'/dm) AND [adult]/lim AND 'article'/it
	#9	#7 AND #8
	#8	#2 OR #3 OR #6
	#7	#1 AND #4 AND #5
	#6	polydioxanone:ti,ab,kw
	#5	'visual analogue scale score':ti,ab,kw OR vas:ti,ab,kw OR 'weight bearing pain':ti,ab,kw
	#4	sham:ti,ab,kw OR placebo:ti,ab,kw OR 'saline control':ti,ab,kw
	#3	polynucleotide:ti,ab,kw OR pn:ti,ab,kw
	#2	'sodium hyaluronate':ti,ab,kw OR 'hyaluronate acid':ti,ab,kw OR hyaluronate:ti,ab,kw OR hyaluronan:ti,ab,kw
	#1	osteoarthritis:ti,ab,kw OR osteoarthrosis:ti,ab,kw OR arthritis:ti,ab,kw OR arthrosis:ti,ab,kw OR gonarthritis:ti,ab,kw OR 'knee near arthralgia':ti,ab,kw

Supplementary Table S2. Search strategies

Cochran	#1	(Osteoarthritis):ti,ab,kw OR (Osteoarthrosis):ti,ab,kw (Word variations have been searched)
	#2	(arthritis):ti,ab,kw OR (arthrosis):ti,ab,kw (Word variations have been searched)
	#3	(Gonarthritis):ti,ab,kw (Word variations have been searched)
	#4	(knee near arthralgia):ti,ab,kw (Word variations have been searched)
	#5	#1 or #2 or #3 or #4
	#6	(Sodium hyaluronate):ti,ab,kw OR (Hyaluronate acid):ti,ab,kw OR (Hyaluronate):ti,ab,kw OR (Viscosupplementation):ti,ab,kw OR (hyaluronan):ti,ab,kw (Word variations have been searched)
	#7	(polynucleotide):ti,ab,kw OR (pn):ti,ab,kw (Word variations have been searched)
	#8	(sham):ti,ab,kw OR (placebo):ti,ab,kw OR ("saline control"):ti,ab,kw (Word variations have been searched)
	#9	("visual analogue scale score"):ti,ab,kw OR (vas):ti,ab,kw AND (weight bearing pain):ti,ab,kw (Word variations have been searched)
	#10	(Polydioxanone):ti,ab,kw (Word variations have been searched)
	#11	#6 or #7 or #10
	#12	#5 and #9 and #8 and #11
KoreaMed	#10	(((((((((osteoarthritis[TIAB] OR osteoarthrosis[TIAB]) OR arthritis[TIAB]) OR arthrosis[TIAB]) OR gonarthritis[TIAB]) OR "'knee near arthralgia'"[TIAB]) AND ((sham[TIAB] OR placebo[TIAB]) OR "'saline control'"[TIAB])) AND (("'visual analogue scale score'"[TIAB] OR vas[TIAB]) OR "'weight bearing pain'"[TIAB]))) AND (((polydioxanone[TIAB]) OR ((polynucleotide[TIAB] OR pn[TIAB])) OR (((("sodium hyaluronate"[TIAB] OR "hyaluronate acid"[TIAB]) OR hyaluronate[TIAB]) OR hyaluronan[TIAB])))))
	#9	((polydioxanone[TIAB]) OR ((polynucleotide[TIAB] OR pn[TIAB])) OR (((("sodium hyaluronate"[TIAB] OR "hyaluronate acid"[TIAB]) OR hyaluronate[TIAB]) OR hyaluronan[TIAB])))
	#8	(((((((osteoarthritis[TIAB] OR osteoarthrosis[TIAB]) OR arthritis[TIAB]) OR arthrosis[TIAB]) OR gonarthritis[TIAB]) OR "'knee near arthralgia'"[TIAB]) AND ((sham[TIAB] OR placebo[TIAB]) OR "'saline control'"[TIAB])) AND (("'visual analogue scale score'"[TIAB] OR vas[TIAB]) OR "'weight bearing pain'"[TIAB]))

Supplementary Table S2. Search strategies

	#7	((((((osteoarthritis[TIAB] OR osteoarthrosis[TIAB]) OR arthritis[TIAB]) OR arthrosis[TIAB]) OR gonarthritis[TIAB]) OR '"knee near arthralgia'"[TIAB]) AND ((sham[TIAB] OR placebo[TIAB]) OR '"saline control'"[TIAB]))
	#6	polydioxanone[TIAB]
	#5	(('"visual analogue scale score'"[TIAB] OR vas[TIAB]) OR '"weight bearing pain'"[TIAB])
	#4	((sham[TIAB] OR placebo[TIAB]) OR '"saline control'"[TIAB])
	#3	(polynucleotide[TIAB] OR pn[TIAB])
	#2	((("sodium hyaluronate"[TIAB] OR "hyaluronate acid"[TIAB]) OR hyaluronate[TIAB]) OR hyaluronan[TIAB])
	#1	(((((osteoarthritis[TIAB] OR osteoarthrosis[TIAB]) OR arthritis[TIAB]) OR arthrosis[TIAB]) OR gonarthritis[TIAB]) OR '"knee near arthralgia'"[TIAB])
