# Supplementary figures and images for: The Efficacy of Polydioxanone Sutures in Treating Mild-to-Moderate Knee Osteoarthritis: A Systematic Review and Meta-Analysis
Source: Medicina (Kaunas). 2025 Feb 24;61(3):388. doi: 10.3390/medicina61030388 (PMC11944242; doi:10.3390/medicina61030388)

## Supplementary Figure S2. Risk of bias graph

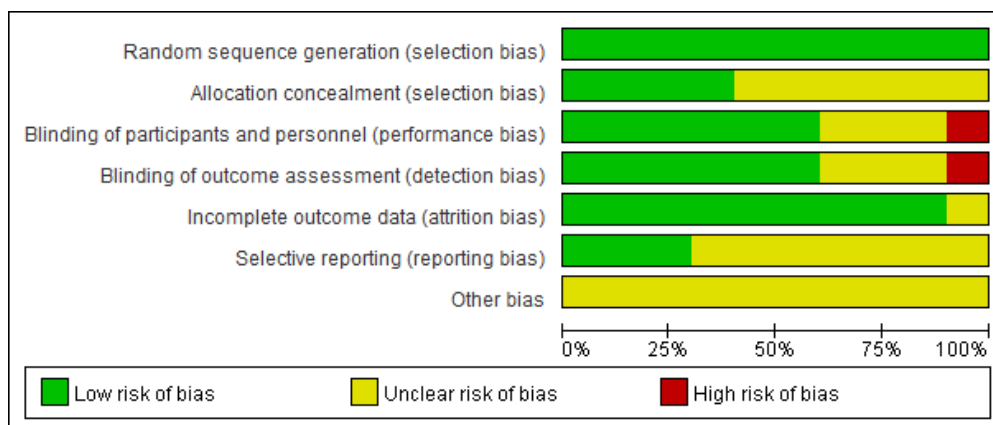

Risk of bias graph (RCT) [39]

Supplement: Supplementary file 1 [file medicina-61-00388-s001.zip › Supplementary Figure S2. Risk of bias graph_24Feb2025.pdf]
